# B Cell Response Is Required for Granuloma Formation in the Early Infection of *Schistosoma japonicum*

**DOI:** 10.1371/journal.pone.0001724

**Published:** 2008-03-05

**Authors:** Fang Ji, Zhanjie Liu, Jianping Cao, Na Li, Zhijian Liu, Jinxin Zuo, Yan Chen, Xinzhi Wang, Jian Sun

**Affiliations:** 1 Shanghai Institute of Immunology, Institutes of Medical Sciences, Shanghai JiaoTong University School of Medicine, Shanghai, People's Republic of China; 2 Health Science Institute, Shanghai Institutes for Biological Sciences, Shanghai JiaoTong University School of Medicine, Chinese Academy of Sciences, Shanghai, People's Republic of China; 3 National Institute of Parasitic Diseases, Chinese Center for Disease Control and Prevention, Shanghai, People's Republic of China; The Rockefeller University, United States of America

## Abstract

*Schistosoma* egg-induced liver granuloma is a dynamic inflammatory reaction that results from complex immune responses to the infection. However, the role of B cells in inflammatory granuloma development is not yet fully understood. We report here that B cell function is required for *S. japonicum* egg-induced granuloma pathology in early infection. Both OBF-1 knockout mice and µMT mice develop severely reduced hepatic granulomas at five weeks post-infection compared to their wild-type counterparts. In contrast, they display no significant difference in granuloma pathology at eight weeks post-infection. Moreover, we find that B cells and antibodies accumulate in the granulomas of wild-type mice early in the infection, indicating a contribution of the B cell response to the granulomatous inflammation. Furthermore, defects in B cell function markedly reduce liver egg burden. These results suggest an important role for B cells in early granuloma pathology. Surprisingly, we found that the *S. japonicum* infection destroys the structure of the lymphoid follicles. This disruptive effect is correlated with a severely impaired T cell-dependent antibody response upon challenge with ovalbumin. Thus, these findings reveal a novel aspect of the interaction between *Schistosoma* and the host immune system.

## Introduction

Schistosomiasis, prevalent in tropical and subtropical areas, is a disease caused by parasitic worms. Infection with *Schistosoma* (*S.*) *mansoni, S. haematobium*, or *S. japonicum* causes an illness in humans that is characterized by the formation of inflammatory granulomas around the deposited parasite eggs. The host immune response is critical for the development of this granuloma pathology [1]. A number of studies in genetically modified mouse models have indicated that granuloma formation is CD4^+^ T cell-dependent [2]. Th1 cells play a major role in the early immune response by secreting IFN-γ, and the immune system gradually succumbs to a Th2 cell response that produces IL-4, IL-5, IL-10, and IL-13 [2]–[7]. However, the role of B cell responses in *Schistosoma* egg-induced granuloma pathology has not been thoroughly studied. Previous studies using B cell–deficient mouse models have indicated that B cell function is not required for granuloma formation in response to *Schistosoma* infection or after egg injection [8]–[11].

OBF-1 (also called OCA-B or Bob-1) is a B cell–specific transcriptional co-activator that promotes immunoglobulin gene transcription by interacting with the POU domains of Oct-1 or Oct-2 [12]. Targeted deletion of OBF-1 impairs B cell development and results in a reduced number of peripheral B cells. In OBF-1-null mice, germinal centers (GC) fail to develop in response to an antigen challenge, and these mice display severely reduced production of IgG antibodies to TI and especially TD antigens [13]–[15].

In this study, we used OBF-1-null mice to investigate the role of B cell function in *S. japonicum* egg–induced hepatic granuloma pathology. Although these mice displayed severely impaired B cell responses to the parasite, they ultimately developed granulomas comparable to their wild-type littermates at eight weeks post-infection. Interestingly, OBF-1-deficient mice failed to form granulomas at five weeks after infection. This finding was confirmed in B cell–deficient µMT mice. Unexpectedly, we found that the structure of lymphoid follicles was progressively disrupted in *S. japonicum*-infected mice.

## Results

### OBF-1-deficient mice show severely impaired B cell responses to *S. japonicum*

**Figure 1:**
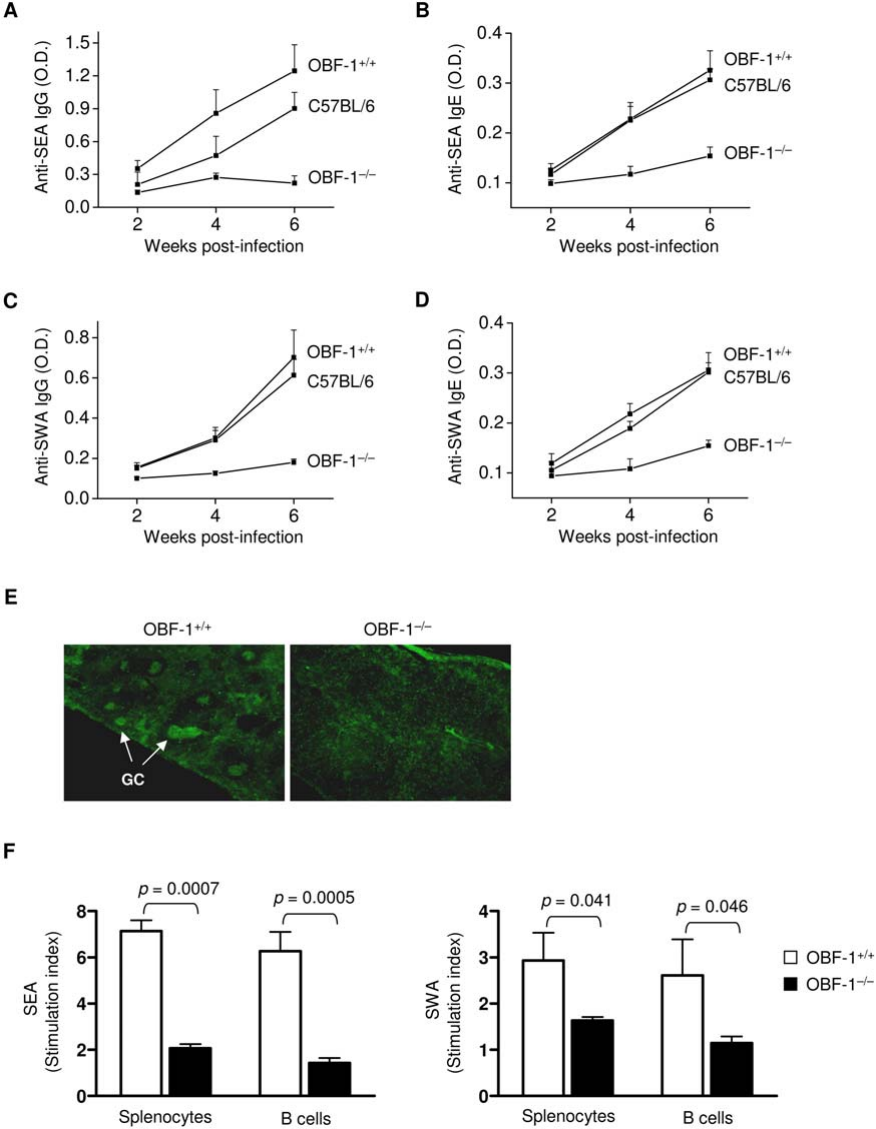
OBF-1-null mice have severely impaired B cell responses to *S. japonicum* infection. (A–D) Sera were collected at the times indicated, and SEA- or SWA-specific IgG (1∶50 dilution) and IgE (1:10 dilution) were measured by ELISA. Results are shown as the means±SD from six mice in each group. (E) Frozen spleen sections prepared from mice at 5 weeks post-infection were stained with biotinylated PNA followed by streptavidin-FITC to reveal GCs (arrow). Six mice in each genotype were examined. Similar results were obtained in each group. Representative pictures are shown. Magnification: 40×. (F) Splenocytes and splenic B cells from mice at 5 weeks post-infection were cultured with or without SEA or SWA for 3 days. Cell proliferation was determined as described in the Materials and Methods. Results represent the mean±SD of four mice for each genotype. Statistically significant differences are shown as *p* values.

To investigate the role of B cells in *S. japonicum* egg-induced granulomatous pathology, we examined B cell responses to the parasite in OBF-1 knockout mice. After infection, OBF-1 wild-type mice displayed serum levels of SEA-specific IgG that increased with time after infection (Fig. 1A). A similar result was observed in C57BL/6 mice. In contrast, OBF-1-null mice did not produce significant quantities of IgG against SEA at any of the time points (Fig. 1A). Similarly, the level of serum SEA-specific IgE was markedly reduced in the OBF-1-null mice compared to littermate controls or C57BL/6 mice (Fig. 1B). We next measured the production of antibodies to SWA in infected OBF-1-null mice. Consistent with the results for SEA-specific antibodies, the levels of both SWA-specific IgG (Fig. 1C) and IgE (Fig. 1D) were markedly decreased in OBF-1-null mice compared to wild-type controls. These data indicate that OBF-1 is critical for the antibody response to *S. japonicum* infection.

GCs are required for antibody production [16]. To determine if defective GC development underlies the failure to mount an antibody response to *S. japonicum* infection in OBF-1-null mice, we analyzed splenic GC formation following infection. Whereas OBF-1 wild-type mice mount a GC response to *S. japonicum* at five weeks post-infection, no GCs were observed in OBF-1-deficent mice (Fig. 1E). This finding is consistent with previous observations [13]–[15]. We then isolated splenic B cells from mice at five weeks post-infection and analyzed their proliferative activity in response to *S. japonicum* antigens. OBF-1-deficent B cells displayed a markedly impaired proliferation response to both SEA and SWA (Fig. 1F). Similar results were also observed in the splenocytes of OBF-1-deficent mice. Together, these results demonstrate that OBF-1-deficent mice have severely impaired B cell responses to *S. japonicum* infection.

### OBF-1-deficient mice fail to develop *S. japonicum* egg–induced granulomas early in infection

**Figure 2:**
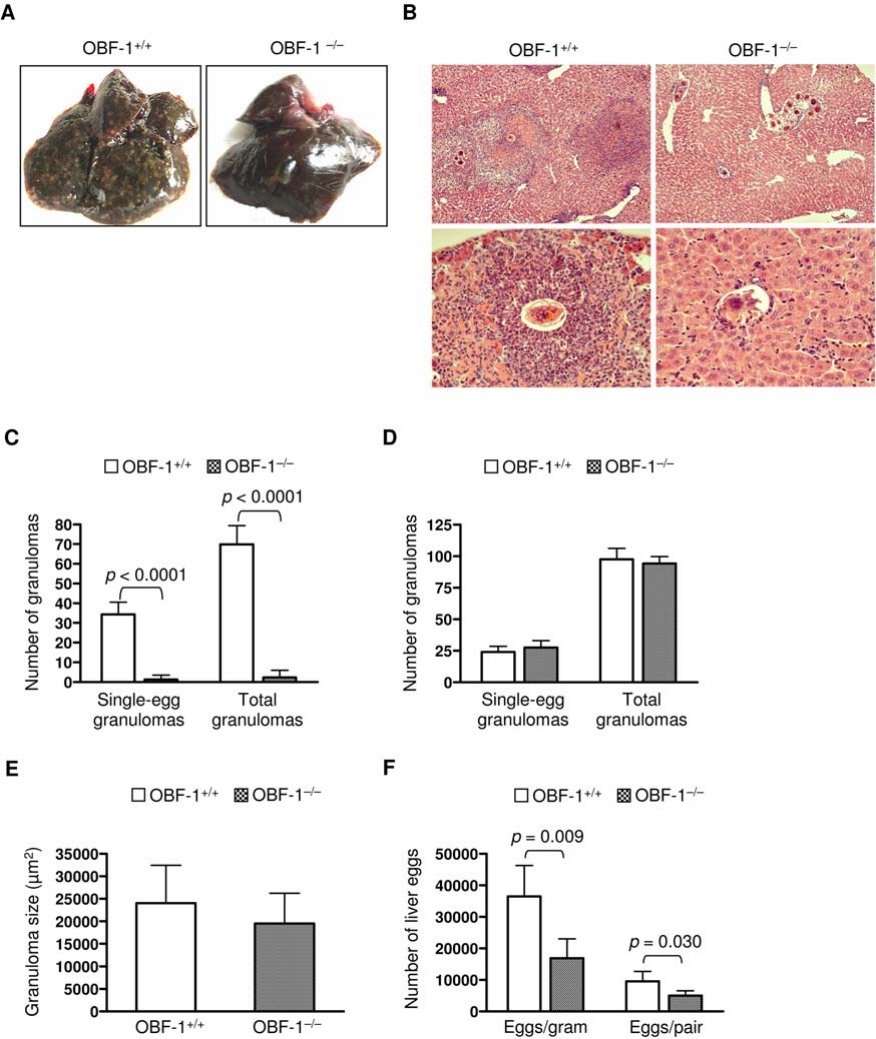
OBF-1-deficient mice fail to develop hepatic granulomas early in *S. japonicum* infection. (A) OBF-1 wild-type or null mice (seven mice for each genotype) were analyzed for granuloma pathology at 5 weeks after *S. japonicum* infection. Representative pictures of a liver from each genotype are shown. (B) Paraffin-embedded liver sections prepared from mice analyzed in (A) were stained with H&E to reveal granulomas. The images shown for each genotype are of livers from two different mice. Magnification: 100× (upper), 400× (lower). (C) The number of granulomas containing a single *S. japonicum* egg or the total number of granulomas in 30 optical fields (100×) of paraffin-embedded liver sections was counted for each mouse. Results are presented as the mean±SD of seven mice for each genotype. *p* values for the compared groups are given. (D) OBF-1 wild-type (n = 6) and null mice (n = 7) were analyzed for granuloma pathology at 8 weeks post-infection. The number of liver granulomas was analyzed as in (C). (E) Liver eggs from seven mice in each genotype were analyzed at 5 weeks post-infection and expressed as egg number per gram liver or female worm. Bars indicate the mean±SD. Statistically significant differences between the compared groups are shown as *p* values.

In mice, granulomas progressively develop after the deposition of *S. japonicum* eggs in the liver, and they reach a peak at about eight weeks post-infection [2]. To determine if granuloma development in OBF-1-null mice proceeds with the same kinetics as in wild-type mice, we analyzed liver granuloma pathology at both five and eight weeks after *S. japonicum* infection. The liver weights of OBF-1-deficient mice were significantly lower than those of their wild-type littermates at five weeks post-infection (Table 1). Many hepatic surface nodes, which are indicative of granuloma pathology, were observed in the wild-type mice; such nodes were not observed on the livers of the OBF-1-deficient mice at the same stage of infection (Fig. 2A). Consistent with these observations, H&E staining of paraffin-embedded liver sections showed that *S. japonicum* eggs induced extensive granuloma formation in OBF-1 wild-type mice. However, OBF-1-deficient mice failed to develop granulomas (Fig. 2B). In fact no granulomas were observed in liver sections from four of the seven OBF-1-null mice examined. Quantification of the hepatic granulomas revealed that the OBF-1-null infected mice developed approximately 30-fold fewer single-egg granulomas and had a similar reduction in the total number of granulomas at five weeks post-infection (Fig. 2C).

**Table 1:** Comparison of organ weight between OBF-1^+/+^ and OBF-1^−/−^ mice.

	5 weeks		*p* value	8 weeks		*p* value
	OBF-1^+/+^ (n = 7)	OBF-1^−/−^ (n = 7)		OBF-1^+/+^ (n = 6)	OBF-1^−/−^ (n = 7)	
Liver weight (% body weight)	7.59±0.75	6.53±0.58	0.021	8.41±0.78	10.36±1.59	0.020
Spleen weight (% body weight)	1.23±0.11	0.49±0.04	<0.0001	1.64±0.1	1.51±0.26	0.285

Data shown represent mean±SD

In contrast to what we observed at five weeks post-infection, OBF-1-null mice developed many hepatic surface nodes by week eight of infection. The number of granulomas in the livers of OBF-1-deficient mice was similar to that of wild-type controls (Fig. 2D). Computer image analysis of single-egg granuloma areas showed no significant difference in the size of the granuloma areas between the OBF-1 knockout mice and the controls (Fig. 2E). These results are consistent with previous reports using B cell-deficient µMT and JHD mice [8]–[11]. The livers of OBF-1-deficient mice became enlarged at this stage of infection, and the ratio of liver weight to body weight was larger than that for wild-type control mice (Table 1).

We next examined the burden of adult worms and eggs at five and eight weeks post-infection. No significant difference in the quantity of female worms or female/male pairs was observed between the wild-type and knockout groups at either stage of infection (data not shown). However, OBF-1 deficiency was associated with a significantly reduced burden of eggs in the livers of five week post-infection mice compared to their littermate controls (Fig. 2F). The egg burden was not significantly different at eight weeks post-infection (data not shown).

### Impaired granuloma formation early in infection is caused by a defective B cell response

**Figure 3:**
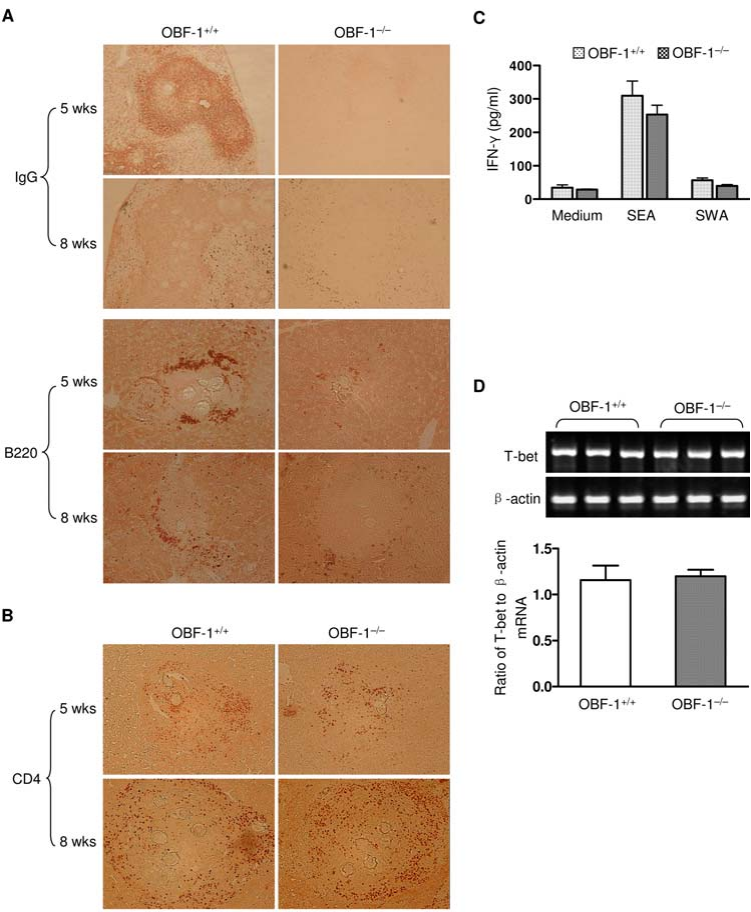
Defective granuloma pathology in OBF-1-null mice is associated with an altered accumulation of IgG and B220^+^ cells but not changes in the CD4^+^ or Th1 cell responses. (A) IgG deposition and B cell accumulation in the liver granulomas of OBF-1 wild-type and null mice was assayed at 5 or 8 weeks post-infection by immunohistochemical staining. Six or seven mice of each genotype were analyzed, and representative images are shown. Magnification: IgG (upper): 100×; B200 (lower): 200×. (B) CD4^+^ cell accumulation in liver granulomas was analyzed in six mice for each experimental condition. Representative images are shown. Magnification: 200×. (C) Splenocytes from 5 week post-infection mice were cultured with or without SEA or SWA for 7 days. Supernatants were collected, and the concentration of IFN-γ was determined by ELISA. Bars show the mean±SD of four mice for each genotype. (D) The mRNA expression of T-bet in splenic cells was measured by RT-PCR (upper). β-actin was used as an internal control. Relative T-bet mRNA levels are presented as a mean±SD (lower).

Accumulation of cells and molecules in an inflamed site could affect the development of the inflammatory reaction. To further probe the role of B cell function in granuloma pathology, we performed immunohistological staining of infected liver sections with anti-IgG and anti-B220 antibodies. A massive deposition of IgG and an accumulation of B cells were evident in the areas of the hepatic granulomas in control mice early in the infection (Fig. 3A). In contrast, no significant deposition of IgG and very few B cells were observed in the livers of OBF-1-null mice at the same point in the infection (Fig. 3A). These data suggest that a deficient B cell response is correlated with the severely impaired granuloma formation observed in OBF-1-deficient mice. Interestingly, we found that antibody deposition and B cell accumulation in granulomas of wild-type mice were markedly reduced at eight weeks compared to five weeks post-infection (Fig. 3A). This observation strongly suggests that the B cell response is more crucial in granulomatous inflammation early in *Schistosoma* infection than late in infection.

We next asked if CD4^+^ cells were involved in the failed granuloma development observed early in the infection of OBF-1-deficient mice. Staining of CD4^+^ cells in the granulomatous sites revealed no significant difference in CD4^+^ cell accumulation between wild-type and knockout mice early in the infection (Fig. 3B). Notably, more CD4^+^ cells were observed in granulomas of both wild-type and OBF-1-null mice at eight weeks post-infection than at five weeks post-infection (Fig. 3B). This observation suggests that CD4^+^ cells could contribute to the development of inflammatory granulomas at eight weeks post-infection in the OBF-1 knockout mice.

We next examined the Th1 response of the OBF-1 knockout mice by measuring the amount of IFN-γ secreted into the supernatant of splenic cell cultures from OBF-1-null or littermate control mice. IFN-γ levels in supernatants of splenic cells cultured in the presence of either SEA or SWA were comparable between OBF-1-null and wild-type control mice (Fig. 3C). Furthermore, the mRNA expression of the transcription factor T-bet, which is critical for the Th1 response [17], was not significantly different between the OBF-1 knockout mice and control mice (Fig. 3D). These results indicate that the Th1 response is not significantly affected in the OBF-1 knockout mice.

**Figure 4:**
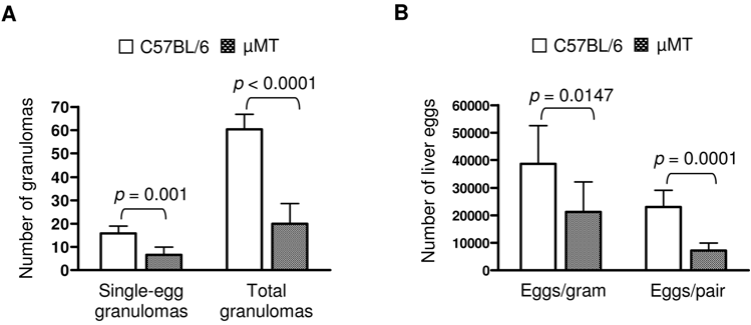
B cell-deficient µMT mice also show markedly impaired granuloma formation at 5 weeks post-infection. (A) Liver granulomas from µMT (n = 9) and C57BL/6 (n = 7) mice were analyzed as described in Fig. 2C. Bars indicate the mean±SD and *p* values for the compared groups are shown. (B) Liver eggs from mice analyzed in (A) were counted. Data are presented as the mean±SD, and the *p* values are given.

To confirm the role of B cells in the egg-induced granuloma formation in early infection, we studied granuloma development in response to *S. japonicum* in B cell-deficient µMT mice. *S. japonicum*-infected µMT mice displayed many fewer hepatic surface nodes than control C57BL/6 mice at five weeks post-infection (data not shown). Quantitative analysis of granuloma formation at five weeks post-infection revealed that the µMT mice developed markedly fewer granulomas in their livers (Fig. 4A). Moreover, the burden of liver eggs in µMT mice was significantly reduced at this stage of infection (Fig. 4B). These data are consistent with our observations in OBF-1-null mice. Together, our observations in these two mouse models indicate that a B cell response is required for the development of granulomas early in *Schistosoma* infection.

### *S. japonicum* infection destroys the structure of the lymphoid follicles

Splenomegaly is characteristic of *Schistosoma* infection. At five weeks post-infection, the spleens of wild-type mice were markedly enlarged. At this point in the infection, the relative weights of the spleens of wild-type mice were significantly greater than those of OBF-1-deficient mice (Table 1). By eight weeks, however, the spleens of OBF-1-null mice had become enlarged. The relative spleen weights at this time were similar to those of wild-type mice.

**Figure 5:**
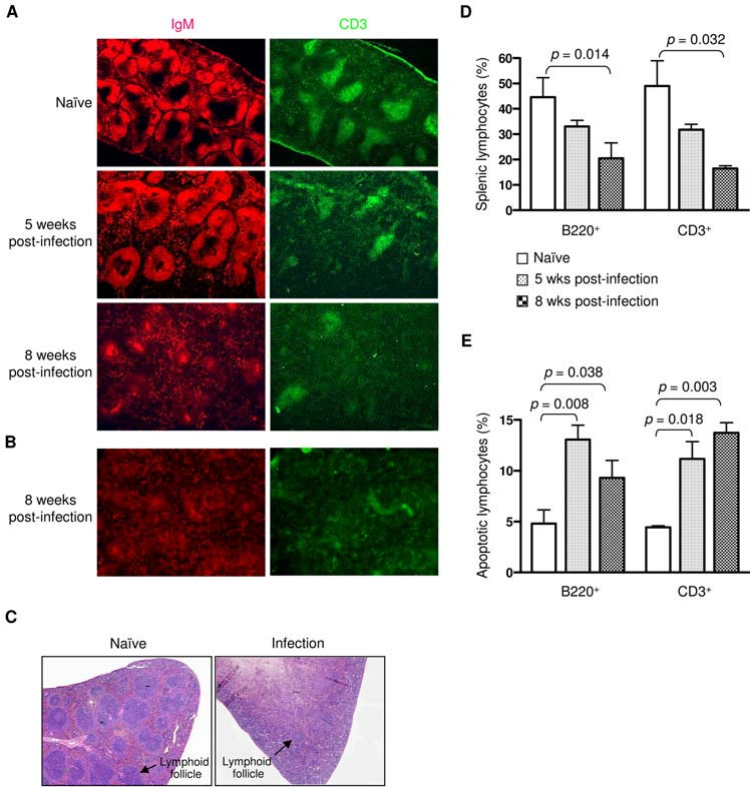
*S. japonicum* infection disrupts the structure of the splenic lymphoid follicles. (A, B) Splenic cryosections were stained with anti-IgM-rhodamine and anti-CD3-FITC to reveal the follicle B zone (red) and T zone (green), respectively. Six OBF-1 wild-type mice (A) and three C57B/6 mice (B) were analyzed in each experiment. Representative images are shown. Magnification: 40×. (C) Paraffin-embedded spleen sections prepared from C56BL/6 mice of 8 weeks post-infection were stained with H&E to reveal lymphoid follicles. Naïve mice were used as controls. Three mice in each group were analyzed. Magnification: 40×. (D) Splenic cells from naïve or infected OBF-1 wild-type mice (six mice for each group) were stained with anti-B220 or anti-CD3. Splenic B220^+^ or CD3^+^ cells were analyzed by flow cytometry. (E) Splenic cells from these mice (six mice for each group) were stained with Annexin V plus anti-B220 or anti-CD3. Apoptotic splenic B220^+^ or CD3^+^ cells are shown. Results are presented as a mean±SD. Statistically significant differences between the compared groups are given as *p* values.

The lymphoid follicle, which consists of a B zone (also called the B cell follicle) and a T zone, is an important site for the development of both lymphocytes and an immune response [18]–[20]. To define the status of splenic lymphoid follicles during *S. japonicum* infection, we stained frozen splenic sections with anti-IgM or anti-CD3 antibodies to reveal the B zone and T zone, respectively. We found that the parasite-infected wild-type mice showed a reduced number of lymphoid follicles at five weeks post-infection compared with naïve mice (Fig. 5A). However, the structure of the lymphoid follicle was largely intact and similar to that of the naïve mice.

Unexpectedly, as the infection progressed, the lymphoid follicles became severely disrupted and were poorly defined by eight weeks post-infection (Fig. 5A). This finding was further confirmed by examination of *S. japonicum*–infected C57BL/6 mice (Fig. 5B and C). We observed similar results in OBF-1-deficent mice; however, the degree of disruption of B cell follicles was less than that of wild-type mice (Supporting information, Fig. S1). We also noted severe disruption in the lymphoid follicles of mesenteric lymph nodes in the wild-type mice (data not shown). These data indicate that *Schistosoma* infection dramatically disrupts the host immune system.

We next analyzed the effect of *Schistosoma* infection on splenic B and T cells using flow cytometry. At five weeks of infection, the number of splenic B (B220^+^) and T (CD3^+^) cells was reduced in comparison to naïve mice (Fig. 5D). Further reduction in these cells was observed at eight weeks post-infection, indicating that infection by *S. japonicum* reduces splenic lymphocytes. This is consistent with the finding that splenic lymphoid follicles are destroyed as the *S. japonicum* infection progresses.

To determine the mechanisms by which splenic lymphocytes are reduced in *S. japonicum*-infect mice, we stained splenic cells with Annexin V and anti-B220 or anti-CD3. Flow cytometry of the stained cells revealed that the proportions of Annexin V-positive B and T cells were significantly increased in infected mice compared to naïve mice (Fig. 5E). These results indicate that the *S. japonicum* infection induces splenic B and T cell apoptosis, which may contribute to the lymphocyte reduction and destruction of lymphoid follicles.

**Figure 6:**
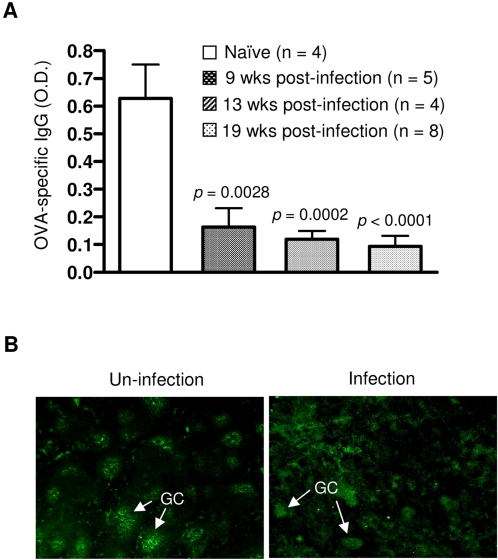
*S. japonicum* infection impairs the T-cell dependent antibody response. (A) Naïve or infected C57BL/6 mice were injected with 50 µg of OVA. Sera were collected at 12 days after OVA immunization. OVA-specific IgG in sera (1:50 dilution) was measured by ELISA. Data are presented as a mean±SD. *p* values are shown for the comparison between infected mice at each time point and the uninfected control group. (B) C56BL/6 mice, either uninfected or at 8 weeks post-infection, were challenged with OVA for 12 days. Frozen splenic sections were stained with biotinylated PNA followed by streptavidin-FITC to reveal GCs. Magnification: 40×.

To investigate the effect of lymphoid follicle disruption on the immune response, we challenged *Schistosoma*-infected C57BL/6 mice with the T cell-dependent antigen OVA and analyzed the development of anti-OVA antibody. Each of the infected mice developed markedly reduced levels of an OVA-specific IgG antibody compared to the controls (Fig. 6A). We next examined GC formation upon challenge with OVA and found that C57BL/6 mice mounted an impaired GC formation at eight weeks post-infection (Fig. 6B). This is consistent with the inability of *Schistosoma*-infected C57BL/6 mice to produce an OVA-specific IgG antibody. Together, these data suggest that the gross disruption of the lymphoid follicles is correlated with a reduction in both B and T lymphocytes through apoptosis induced by the *S. japonicum* infection.

## Discussion

*Schistosoma* egg-induced liver granuloma represents a dynamic inflammatory reaction that results from complex immune responses to the infection. The role of B cells in *Schistosoma* egg-induced granuloma formation has not yet been completely defined. Previous studies have shown that B cell-deficient µMT and JHD mice do not have impaired granuloma formation at time points after seven weeks of *S. mansoni* infection or egg injection [8]–[11], suggesting that B cells are not absolutely required for granuloma formation late in infection. To further explore the requirement for B cells in this process, we characterized the response of OBF-1 knockout mice to *S. japonicum* infection. We and others have reported that OBF-1 is critical for B cell responses to multiple antigens and autoantigens [13]–[15], [21], [22]. We found that OBF-1-deficient mice exhibited severely impaired B cell responses to infection with *S. japonicum*. Like µMT and JHD mice, the granuloma pathology of OBF-1-null mice was comparable to that of littermate control mice at eight weeks post-infection [8]–[11]. However, we found that OBF-1 knockout mice have a severe defect in granuloma formation at five weeks after infection. This finding was further confirmed in B-cell deficient µMT mice infected by *S. japonicum*. Thus, these results delineate a previously unrecognized mechanism for host B cell function in the dynamic development of granulomas during *Schistosoma* infection.

B cells function in the immune response through both antibody-dependent and antibody-independent mechanisms. The antibody-independent B cell activities include the secretion of proinflammatory cytokines and chemokines and antigen presentation [23], [24]. The role of B cells and antibody in inflamed tissues is now widely recognized [25], and a recent study reports that functional B cells exist in the liver and contribute to hepatic fibrosis [26]. When we examined sites of inflammation in the livers of mice at five weeks post *S. japonicum*-infection, we observed a robust deposition of antibody and an accumulation of B cells in the granulomas of wild-type mice. At eight weeks post-infection, however, the B cell response had largely disappeared from the granulomas. These results strongly suggest that B cells contribute to the development of inflammatory granulomas in early infection, but are no longer required late in infection.

**Figure 7:**
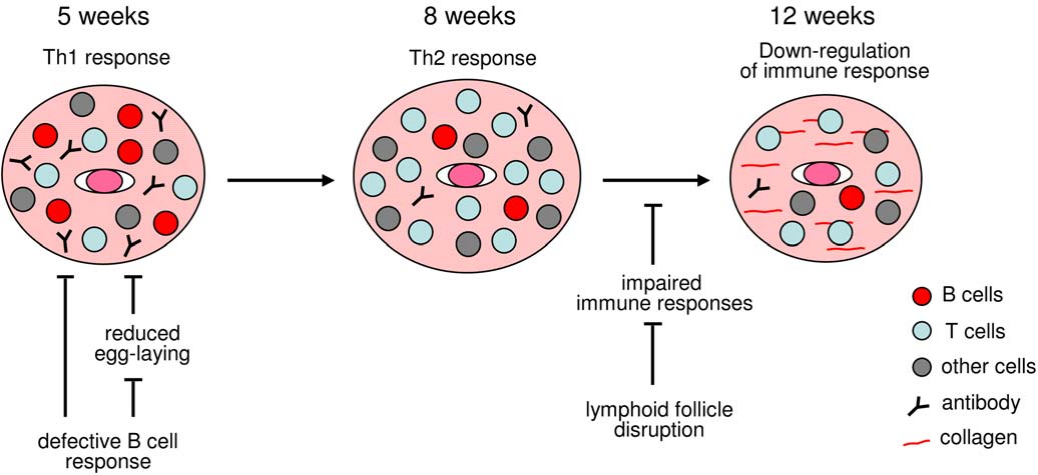
A working model for the function of B cells in granuloma pathology and the effect of lymphoid follicle disruption on down-regulation of the immune response and granulomas. At early stages of *Schistosoma* infection (5 weeks), B cell function is required for granuloma formation. Mice with B cell deficiencies fail to mount an effective inflammatory reaction. Meanwhile, deficient B cell function also affects liver egg burden and produces impaired granuloma pathology. At later stages (7–8 weeks), the Th2 response exerts a dominate effect on granuloma pathology (ref. 2) and is accompanied by both an accumulation of CD4^+^ cells and a reduction of B220^+^ cells and antibody in granulomas. Infection by *Schistosoma* destroys the lymphoid follicles at 8 weeks and degrades immune responses, such as the T helper cell-dependent B cell response to OVA. Finally, granuloma pathology diminishes after 12 weeks of infection.

In the early stages (between three and five weeks) of infection, a Th1 response is mounted [2]. The Th2 response emerges between five and six weeks and peaks at eight weeks post-infection. We found that OBF-1 knockout mice have no significant reduction in the Th1 cell response to *S. japonicum* infection. Furthermore, the Th1 cell response also appears to be normal in the µMT mice [9]. These findings suggest that the impaired granuloma development observed early in the infection of OBF-1-deficient and µMT mice is not likely due to an effect on the Th1 cell response. We also examined the accumulation of CD4^+^ cells in granulomas of OBF-1 knockout mice and found that it is comparable to that of their wild-type controls. CD4^+^ cells accumulate in the inflamed sites as the infection progresses in both the wild-type and knockout mice. Therefore, we propose that a B cell response is required for the development of granulomatous inflammation at the early stages of infection. After this initial phase of granuloma development, a CD4^+^ cell response begins to exert a dominant effect on the pathology and the requirement for B cells is alleviated. As a result, inflammatory granulomas can develop even when the B cell response is defective (Fig. 7).

Our results clearly show that a B cell deficiency is associated with a significant reduction in the liver egg burden at five weeks post-infection. This effect does not appear to be correlated with the worm burden or the male/female pair abundance at this stage of infection. In line with this, it has been reported that the development of *S. mansoni* is not affected in B cell-deficient mice [10], [27]. However, egg excretion is significantly reduced in µMT mice [10]. Our observation that the egg burden is reduced in both OBF-1-deficient mice and µMT mice could reflect an effect of a B cell deficiency on egg-laying during early infection. A B cell response shifts to a CD4+ cell response as the infection progresses, and worm fecundity in B cell-deficient mice becomes comparable to that in B cell-intact mice. The defective B cell response, which results in a delay in egg-laying, could contribute to the marked impairment of granuloma pathology observed in these two mouse models (Fig. 7).

Surprisingly, we found that infection with *S. japonicum* progressively disrupted the structure of the lymphoid follicles. This observation was correlated with both the apoptosis of B and T cells and a lymphocyte reduction following *S. japonicum* infection. Lymphoid follicles are important for the development and functional maturity of lymphocytes and other immune cells, such as follicle dendritic cells and macrophages. These follicles are also essential for mounting an efficient immune response [28]–[32]. To determine if the disruption of lymphoid follicles by *S. japonicum* infection produced impaired immune responses, we investigated the response to the T cell-dependent antigen OVA in *S. japonicum*-infected mice. We found that both GC formation and IgG antibody production in response to OVA were markedly impaired in *S. japonicum*-infected mice. These findings reveal a critical effect of *Schistosoma* on the host immune system and provide structural evidence for understanding and explaining both the immune response to the parasite and its pathology.

Granuloma pathology is down-regulated during the late stage of infection [1], [33]. This is associated with down-regulation of the host immune response to *Schistosoma* late in the infection [33]–[36]. However, the mechanisms mediating the down-regulation of the immune response and the granuloma pathology remain largely unknown. Our findings indicate that *Schistosoma* can affect lymphoid follicles as early as five weeks after infection, and severe disruption of lymphoid follicles was observed at eight weeks post-infection. These progressive effects impair the immune responses to *Schistosoma* and may underlie the diminishing granulomatous inflammation in the chronic stage of infection (Fig. 7). As lymphoid follicles are gradually disrupted, the granulomatous inflammation progresses and peaks at eight weeks post-infection. This could be reflected by a time difference between the follicle destruction in lymphoid tissue and the granuloma development in liver. Additionally, residual lymphocytes and other immune cells in the periphery could contribute to the development of the inflammatory pathology.

Like many helminth parasites, *Schistosoma* efficiently avoids attack by the host immune system and can survive for years in its host. Our findings are suggestive of some mechanisms by which this parasite evades the immune response, and they prompt us to consider the following questions: Does *Schistosoma* escape from immune surveillance and obtain long-term persistence partially through its disruptive effect on the immune system? Does *Schistosoma* induce apoptosis or affect the maturation of memory lymphocytes and thus acquire the ability to reinfect its host? Experimental investigation of these questions will yield important insight into the pathogenesis of schistosomiasis.

## Materials and Methods

### Mice and parasites

OBF-1 knockout mice [13], [37] were maintained under pathogen-free conditions. µMT and C57BL/6 mice were purchased from the Jackson Laboratory (Bar Harbor, ME). Mice were infected percutaneously with 20 cercariae of *S. japonicum* and sacrificed at five or eight weeks after infection for analysis of parasite burden. To analyze parasite egg burden, liver samples were weighed and digested in 4% potassium hydroxide and the number of eggs released was quantified by light microscopy. Adult worms were enumerated following perfusion of the mouse liver. All animal studies were approved by the Institutional Animal Care and Use Committee.

### Preparation of antigens

Soluble egg antigen (SEA) and soluble worm antigen (SWA) were prepared as described previously [38]. The protein concentration of SEA and SWA was determined using the BCA Protein Assay kit (Bio-Rad, Richmond, CA).

### Detection of serum antibodies

Serum levels of SEA- or SWA-specific IgG or IgE and ovalbumin (OVA)-specific IgG were determined by ELISA. Briefly, 96-well plates were coated overnight at 37°C with 10 g/ml SEA, SWA, or OVA. After washing and blocking, serum samples or serum dilution buffer were added. Serum IgG or IgE binding to the plates was measured with HRP-conjugated anti-mouse IgG or IgE, respectively. Color development was determined by measuring the absorbance at 450 nm. All antibodies were purchased from Southern Biotechnology Associates.

### Immunohistochemistry

Lymphoid follicles and GCs were analyzed as described [39]. Briefly, frozen spleen and mesenteric lymph node sections were stained with anti-IgM-rhodamine (Jackson ImmunoResearch Laboratories, West Grove, PA, USA), anti-CD3-FITC (Southern Biotechnology Associates, Birmingham, AL), or peanut agglutinin (PNA)-biotin (Vector Laboratories, Burlingame, CA). Biotinylated PNA was detected with streptavidin-FITC (Southern Biotechnology Associates). Paraffin-embedded liver sections were analyzed for antibody deposition and lymphocyte accumulation. After deparaffinization and quenching of endogenous peroxidase activity, the sections were stained with biotinylated anti-IgG, anti-B220, or anti-CD4 followed by streptavidin-HRP (Southern Biotechnology Associates). 3-Amino-9-ethyl carbazol (AEC) was used as the substrate for color development.

### Proliferation assays

Single-cell suspensions of spleen were routinely prepared. Splenic B cells were isolated (purity >95%) with a B-cell negative isolation kit (Miltenyi Biotec, Germany). *S. japonicum*–infected splenocytes or isolated B cells (1×10^6^ cells/ml) were cultured for three days in RPMI 1640 medium containing 10% FBS and 20 µg/ml SEA or SWA. WST-8 solution was added for the last four hours of cell culture according to the protocol of the Cell Counting Kit-8 (Beyotime, Shanghai, China). Color development was determined by measuring absorbance at 450 nm.

### Assessment of egg pathology

Paraffin-embedded liver sections were stained with hematoxylin and eosin (H&E). The number of granulomas in 30 optical fields (100× magnification) was counted for each mouse. For each mouse, the sizes of 30 granulomas around single eggs were quantified with the KS400 Image Analysis System (Zeiss, Germany). Data are expressed in area units.

### Cytokine assay and RT-PCR

*S. japonicum*–infected splenocytes (1×10^6^ cells/ml) were cultured for seven days in RPMI 1640 medium containing 10% FBS and 20 µg/ml SEA or SWA. Supernatants were harvested and used to analyze the level of cytokines. IFN-γ was measured using commercially available kits (R&D, Minneapolis, MN). mRNA expression of splenic T-bet was analyzed using RT-PCR. Total RNA was extracted with TRIzol (Gibco/BRL, Gaithersburg, USA), and cDNA was prepared with the First-Strand cDNA Synthesis kit (Promega, Madison, WI). The following primers were used in PCR: T-bet: 5′-TGC CTG CAG TGC TTC TAA CA-3′; 5′-TGC CCC GCT TCC TCT CCA ACC AA-3′; β-actin: 5′-TGT TAC CAA CTG GGA CGA CA-3′; 5′-TCT CAG CTG TGG TGG TGA AG-3′.

### Flow cytometry

Splenic cells were stained with anti-B220-APC or anti-CD3-PE (Southern Biotechnology Associates) in combination with Annexin V-FITC (BD PharMingen, San Diego, CA). Stained cells were analyzed using a FACSCalibur cytometry (Becton Dickinson, Mountain View, CA).

### Statistical analysis

Statistical differences were evaluated by the Student's two-tailed *t* test using GraphPad Prism software (San Diego, CA, USA). A *p*<0.05 was considered to be statistically significant.

## Supporting Information

Figure S1 *S. japonicum* infection disrupts the structure of splenic lymphoid follicles in OBF-1 knockout mice. Splenic frozen sections were stained with anti-IgM-rhodamine and anti-CD3-FITC to reveal the follicle B zone (red) and T zone (green), respectively. Six OBF-1 knockout mice were analyzed in each time point. Representative images are shown. Magnification: 40×.
(6.05 MB TIF)
