# Supplementary figures and images for: B Cell Response Is Required for Granuloma Formation in the Early Infection of Schistosoma japonicum
Source: PLoS One. 2008 Mar 5;3(3):e1724. doi: 10.1371/journal.pone.0001724 (PMC2248706; doi:10.1371/journal.pone.0001724)

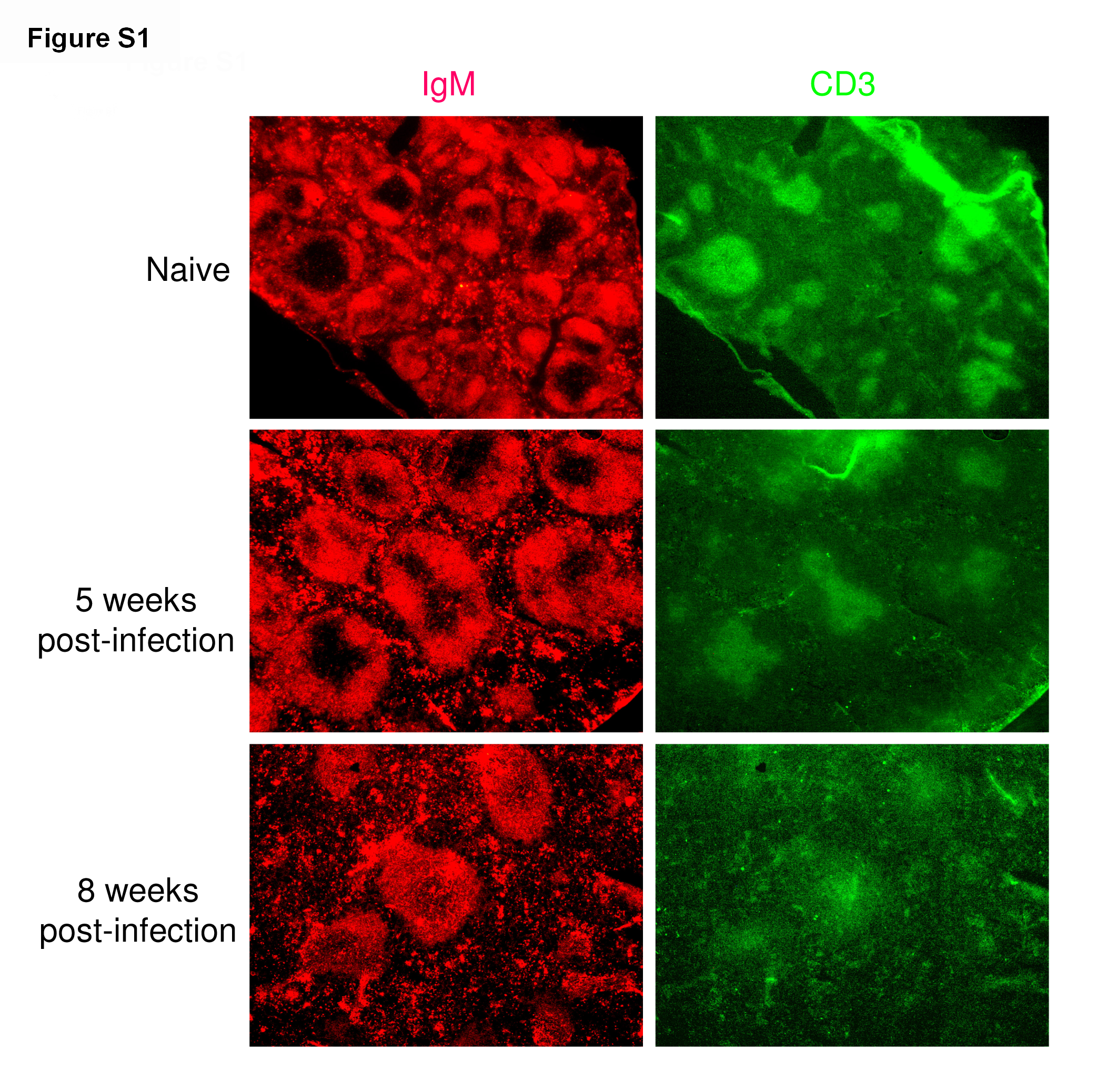

Supplement: Figure S1 — S. japonicum infection disrupts the structure of splenic lymphoid follicles in OBF-1 knockout mice. Splenic frozen sections were stained with anti-IgM-rhodamine and anti-CD3-FITC to reveal the follicle B zone (red) and T zone (green), respectively. Six OBF-1 knockout mice were analyzed in each time point. Representative images are shown. Magnification: 40×. (6.05 MB TIF) [file pone.0001724.s001.tif]
